# The Inhibitory Effects of Alpha 1 Antitrypsin on Endosomal TLR Signaling Pathways

**DOI:** 10.3390/biom15010043

**Published:** 2025-01-01

**Authors:** Ahmed S. Elshikha, Georges Abboud, Rigena Avdiaj, Laurence Morel, Sihong Song

**Affiliations:** 1 Department of Pharmaceutics, College of Pharmacy, University of Florida, Gainesville, FL 32610, USA; 2 Department of Pathology, Immunology and Laboratory Medicine, University of Florida, Gainesville, FL 32610, USAmorel@uthscsa.edu (L.M.)

**Keywords:** systemic lupus erythematosus (SLE), inflammation, toll-like receptors (TLRs), human alpha-1 antitrypsin (hAAT)

## Abstract

Endosomal toll-like receptors (TLRs) TLR7, TLR8, and TLR9 play an important role in systemic lupus erythematosus (SLE) pathogenesis. The proteolytic processing of these receptors in the endolysosome is required for signaling in response to DNA and single-stranded RNA, respectively. Targeting this proteolytic processing may represent a novel strategy to inhibit TLR-mediated pathogenesis. Human alpha 1 antitrypsin (hAAT) is a protease inhibitor with anti-inflammatory and immunoregulatory properties. However, the effect of hAAT on endosomal TLRs remains elusive. In this study, we first tested the effect of hAAT on TLR9 signaling in dendritic cells (DCs). We showed that hAAT inhibited TLR9-mediated DC activation and cytokine production. Human AAT also lowered the expressions of interferon signature genes. Western blot analysis showed that hAAT reduced the expression of the active form (cleaved) of TLR9 in DCs, indicating a novel mechanism of hAAT function in the immune system. We next tested the effect of hAAT on TLR7/8 signaling. Similar to the effect on TLR9 signaling, hAAT also inhibited R848 (TLR7 and 8 agonist)-induced DC activation and functions and lowered the expressions of interferon signature genes. Our in vivo studies using hAAT transgenic mice also showed that hAAT attenuated R848-induced pathogenesis. Specifically, hAAT completely blocked the R848 induction of germinal center T cells (GC T), B cells (GC B), and plasma cells (GC PCs), as well as T follicular T helper cells (T_FH_), which are all critical in lupus development. These data demonstrated that hAAT inhibited TLR7/8 and TLR9 signaling pathways, which are critical for lupus development. These findings not only advanced the current knowledge of hAAT biology, but also implied an insight into the clinical application of hAAT.

## 1. Introduction

Systemic lupus erythematosus (SLE) is an autoimmune disease characterized by loss of tolerance to self-antigens. The clinical heterogeneity of SLE makes it difficult to treat [1]. Currently, there is no cure for SLE and the treatment options are limited, although some biologics are available. Mounting evidence shows dendritic cells (DCs) and IFN-I play critical roles for initiating and maintaining autoimmunity and disease development in lupus [2,3]. Plasmacytoid dendritic cells (pDCs) that link innate and adaptive immunity are the major source of type 1 interferon (IFN-I) in response to autoantigens in the form of immune complexes. IFN-I can activate monocytes or conventional DCs (cDCS) to produce BAFF, a key survival factor for autoreactive B cells. It has been shown that cytokines produced by activated cDCs from lupus mice enhance B cell proliferation and antibody secretion [4], and suppress regulatory T cell (Treg) differentiation and function [5]. In addition, activated DCs can present autoantigens and induce T cell differentiation by up-regulating MHCII and costimulatory molecules, which allows for the direct modulation of the adaptive immune response. Activated pDCs can also indirectly impact T cells by producing inflammatory cytokines. Therefore, inhibiting DC activation and function (e.g., IFN-I production) has great potential for controlling autoimmunity and SLE development.

Toll-like receptors (TLRs) are pattern recognition receptors that play important roles in activating the innate immune system in response to pathogen-associated molecular patterns (PAMPs) or danger-associated molecular patterns (DAMPs). Increasing evidence showed that endosomal TLRs play important roles in the pathogenesis of autoimmune diseases such as SLE. Unlike other TLRs (TLR1, 2, 4, 5, 6, and 10) that are localized to cell membranes and recognize lipid and proteins, TLR3, 7, 8, and 9 are located in endosomes and recognize nucleic acids [6]. Interestingly, TLR 7, 8, and 9 share similar domain organization. They have a characteristic Z-loop between leucine-rich repeats in the ectodomain. It has been shown that the large ectodomain is proteolytically cleaved to be activated for ligand binding in the endolysosome [7,8,9]. The proteolytic process is mostly at the Z-loop and can involve multiple steps and proteases including cathepsins, asparagine endopeptidase, and furin-like proprotein convertases [10,11]. The complexity of endosomal TLR processing may contribute to their functions in different cell types and physiological conditions. The complexity can also be a major factor leading to the challenge of the therapeutical inhibition of these TLR functions [12].

Human alpha 1 antitrypsin (hAAT) is a serine proteinase inhibitor (Serpin) and has anti-inflammatory and immunoregulatory functions [13]. In addition to inhibiting serine proteinases such as neutrophil elastase (NE), proteinase 3, and cathepsin G, it can also inhibit cysteine proteases including caspase 3 and cathepsin K [14,15]. While the inhibitory effect of hAAT on proteinases significantly contributes to its anti-inflammatory property, it has been shown that hAAT regulates immune system by functions independent from proteinase inhibition. Human AAT can interact with the TNF-α receptor and block TNF-a signaling [16]. Human AAT can directly interact with IL-8, and leukotriene B4 and mediate anti-inflammatory functions [17,18]. The therapeutic potentials of hAAT have been demonstrated in various disease models, including type 1 diabetes [19,20,21,22], arthritis [23,24], lupus [12,25,26], osteoporosis [27,28,29], stroke [30], Graft-versus-host disease (GVHD) [31], and, more recently, Coronavirus disease (COVID-19) [32,33]. It has also been shown that hAAT has anti-aging effects [34,35]. However, the mechanism(s) underlying the therapeutic effects remain elusive. Specifically, the effect of hAAT on TLR signaling requires further investigation.

## 2. Materials and Methods

### 2.1. Animals

The B6.NZMSle1/2/3 (B6.TC) congenic strain has been previously described. C57BL/6 male or female mice were purchased at 8–12 weeks old from Jackson Laboratories (Bar Harbor, ME, USA). The human AAT transgenic (hAAT-tg) mice were developed and maintained at the University of Florida. They were initially generated on NOD background using an hAAT expression cassette flanked with AAV2 inverted terminal repeat sequences (ITRs, rAAV2-CB-hAAT) and crossbred with B6 mice for many generations [26]. All animals were housed in specific pathogen-free (SPF) conditions. For R848 treatment, 12–14-week-old hAAT-Tg, B6, and B6.TC mice were randomly assigned into control or treated groups. Mice were treated topically on the right ear with 100 µL R848 in acetone 3 times (2-day interval). Control mice were treated with 100 µL acetone. The animal study protocol was approved by the institutional animal care and use committee (IACUC) of the University of Florida (UF-IACUC number: 201907848, approved on 5 June 2019).

### 2.2. Cytokine Assays

TNF-α, IL-12, and CXCL-10 levels in cell culture media were quantified using ELISA kits (PeproTech, Rocky Hill, NJ, USA) and following manufacturer’s instructions. IFN-I levels were detected using murine IFN-I sensor B16-Blue^TM^ IFN-α/β cells (InvivoGen, San Diego, CA, USA) as previously described [28].

### 2.3. cDC Preparation and Cell Cultures

Bone marrow cells from B6 mice were cultured with or without 0.1, 0.5, 1, and 2 mg/mL hAAT in complete RPMI 1640 (Corning Cellgro, Manassas, VA, USA) containing 10% fetal bovine serum (FBS) (Thermo Fisher Scientific, Waltham, MA, USA), 2- Mercaptoethanol (Sigma-Aldrich, St. Louis, MO, USA), 10 ng/mL GM-CSF, and 5 ng/mL IL-4 (PeproTech, Cranbury, NJ, USA). On day 3, 50% of the culture medium was replaced with fresh medium containing the same supplements. On day 4, cells were stimulated by adding 1, 5, and 10 µg/mL CpG-ODN 1826 (InvivoGen, San Diego, CA, USA) or 1, 3, and 5 µg/mL R848 for the indicated time points. Cells were harvested at the indicated time points; centrifuges and supernatant were stored at −80 °C for cytokine detection. In gene expression experiments, BM cells were treated with or without 1 mg/mL clinical grade hAAT (Prolastin C, Grifols, Los Angeles, CA, USA) and stimulated with or without 5 µg/mL or 1 µg/mL R848 for 1, 4, and 6 h and, for protein detection, cells were stimulated for 15 min.

### 2.4. Flow Cytometry

Single-cell suspensions were prepared using standard procedures from spleens or harvested DCs. Cells were stained in FACS staining buffer (2.5% FBS, 0.05% sodium azide in PBS). Fluorochrome-conjugated antibodies are as follows: B220 (RA3-6B2), CD11b (M1/70), CD11c (HL3), CD62L (MEL-14), CD95 (Jo2), CD80 (16-10 A1), CD86 (GL1), I-A/I-E (M5/114.15.2), I-A^b^ (AF6-120.1), CD19 (eBio1D3), BCL-6 (K112-91), and CD95 (Jo2), which were purchased from BD Biosciences. CD4 (RM4-5) and CD138 (281-2) were purchased from BioLegend (San Diego, CA, USA). CD4 (GK1.5), CD44 (IM7), CD25 (PC61.5), CD69 (H1.2F3), Foxp3 (FJK-16S), GL-7 (GL-7), and PD-1 (RMP1-30) were purchased from eBioscience (San Diego, CA, USA). Dead cells were excluded with fixable viability dye (eFluor780 or LIVE/DEAD™ Fixable Yellow Dead Cell Stain Kit; Thermo Fisher Scientific, Waltham, MA, USA). Intracellular staining was performed with a fixation/permeabilization kit (eBioscience, San Diego, CA, USA). All samples were acquired on an LSRFortessa flow cytometer (BD Biosciences, San Diego, CA, USA) and analyzed with FlowJo software V10 (Tree Star, Woodburn, OR, USA).

### 2.5. Detection of mRNA Levels

Harvested DCs were lysed in RLT buffer (Qiagen, Germantown, MD, USA) with 2% beta mercaptoethanol (Thermo Fisher Scientific). Total RNA was extracted with the RNeasy Mini Kit (Qiagen) and used for qRT-PCR using the High-Capacity cDNA Reverse Transcription Kit (Thermo Fisher Scientific). SYBR Green Dye (BioRAD, Hercules, CA, USA) was used for quantification on the Bio-Rad CFX connect system. The primers used are shown in Table 1. Gene expression was quantified with the 2^−∆∆**Ct**^ method relative to Ppia (cyclophilin A).

### 2.6. Protein Analyses and Western Blot Analyses

Lysates were prepared from cDCs. Cells in lysis buffer and protease inhibitor cocktail were sonicated for 30 s. Total protein was quantified in supernatants using the QC quantification kit (Fisher scientific). Lysates were normalized to 20–30 μg/μL using a cocktail of DTT/dye solution at a ratio of 1:10 before heat denaturation. Protein lysates were separated by SDS-PAGE and transferred onto a PVDF membrane using the BioRad transblot mini-gel system. After blocking with 5% milk proteins in TBS-T at room temperature, the membrane was incubated serially with primary antibodies (1:1000) against pNF-kB, NF-kB, pIkb, IkB, TLR9, and (1:2000) β-Actin at 4 °C. HRP-conjugated anti-rabbit or anti-mouse IgG were used as secondary antibodies (1:2000 in TBS-T with 5% milk proteins). Signal was detected using Signal-Fire or Signal-Fire-Elite. For repeated measurements of different proteins on the same membrane, PVDF stripping buffer was applied for 15 min and the membrane was re-blocked before re-probing. Band intensities were quantified using ImageJ software.

### 2.7. Statistics

We performed statistical analyses using the Graphpad Prism 9.0 software. Differences between groups were evaluated by one-way ANOVA with multiple comparisons. We also performed *t* tests as indicated in the text. All tests are two-tailed. Results were expressed as means ± standard deviation. The levels of statistical significance were set at *: *p* < 0.05, **: *p* < 0.01, ***: *p* < 0.001, and ****: *p* > 0.0001.

## 3. Results

### 3.1. Human AAT Dose Dependently Inhibits TLR9 Pathway

**Figure 1:**
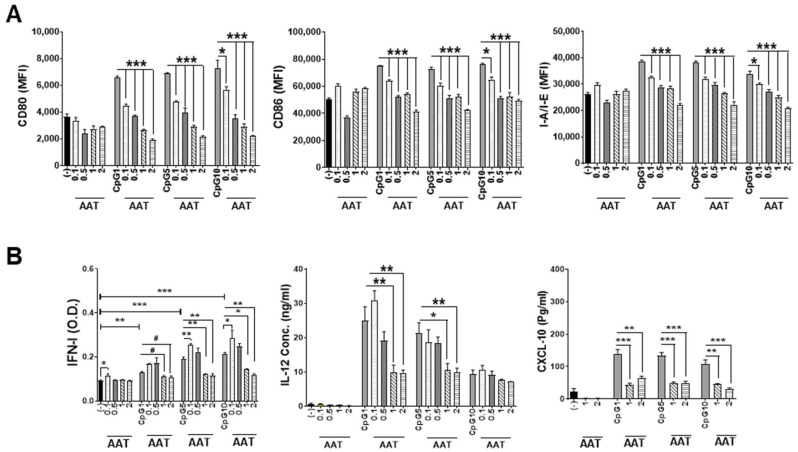
**The effect of hAAT on CpG-induced DC activation and functions**. Conventional DCs were induced from BM cells from B6 mice. During the induction, cells were treated with or without 0.1, 0.5, 1, or 2 mg/mL hAAT. After the induction, cells were stimulated with CpG (0, 1, 5, and 10 µg/mL) for 6 h and subjected to flow cytometry analyses to detect DC activation using antibodies recognizing CD80, CD86, and I-A/I-E (**A**). Cytokines (IFN-I, IL-12, and chemokine CXCL10) in culture media were detected (**B**). n = 3 (cells from 3 mice) for all groups. *, *p* < 0.05; **, *p* < 0.01; and ***, *p* < 0.001 by one-way ANOVA. #, *p* < 0.05 by *t* test.

We have previously show that hAAT treatment inhibited CpG (TLR9 agonist)-induced DC activation [12,25]. To further investigate this effect, we performed a set of experiments with different conditions and tested different doses of hAAT on DC activation. As shown in Figure 1A and Figure S1A–C, hAAT treatment inhibited DC activation in a dose-dependent manner in all the conditions (1, 5 and 10 µg/mL of CpG). Remarkably, low-dose (0.1 mg/mL) hAAT can significantly inhibit DC activation. This effect can be observed as early as 6 h after the treatment. Similarly, hAAT also inhibited DC function (IFN-I, IL-12, and CXCL-10 secretions), although in a relatively higher dose (Figure 1B and Figure S1D–F). These results indicated that DC activation is more sensitive to hAAT treatment than DC functions. We also observed that a low dose (1 µg/mL) of CpG is sufficient for DC activation and increasing the CpG dose did not result in further enhancement (Figure 1A). Interestingly, increasing the CpG dose significantly increased IFN-I production, whereas it decreased IL-12 and CXCL-10 production (Figure 1B).

### 3.2. The Effect of hAAT on Downstream Gene Expressions of TLR9

**Figure 2:**
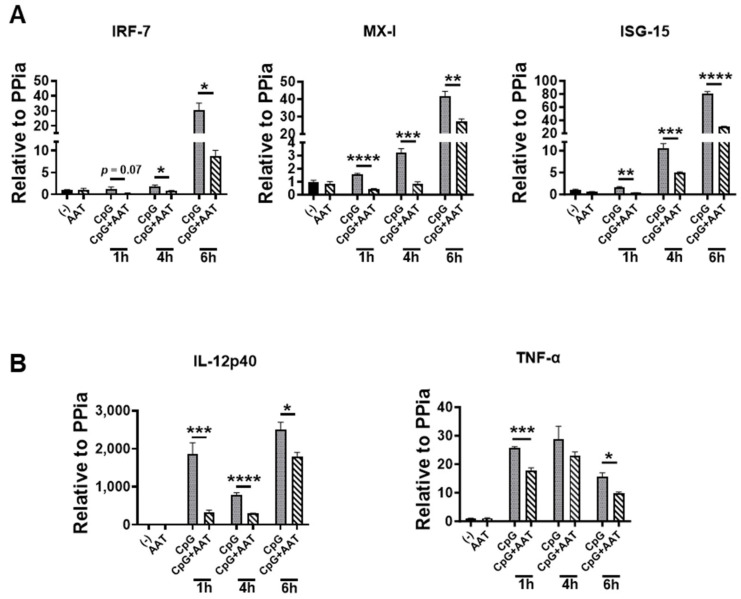
**The effect of hAAT on CpG-induced gene expression.** (**A**). IFN-I signature genes. (**B**). Cytokine genes. DCs treated with or without 1 mg/mL hAAT were stimulated with 0 or 1 μg/mL CpG for 6 h. The cells were harvested and gene expressions were assessed by RT-qPCR using primers specific to the genes of IRF-7, Mx-1, and ISG-15, and IL-12p40 or TNF-α. n = 6 mice for all groups. *, *p* < 0.05; **, *p* < 0.01; ***, *p* < 0.001; and ****, *p* < 0.0001 by *t* test.

To further elucidate the effect of the hAAT inhibition of DC activation, we treated DCs with or without 1 mg/mL hAAT and stimulated with or without TLR9 agonist (CpG). We assessed gene expression in these cells by RT-qPCR. As shown in Figure 2, hAAT treatment significantly reduced the gene expression levels of IFN-I signature genes, IRF-7, Mx-1, and ISG-15 (Figure 2A). Moreover, gene expression levels of IL-12p40 (the active moiety of IL-12) and TNF-α were significantly lowered by hAAT treatment (Figure 2B).

### 3.3. The Effect of hAAT on the Activation of TLR9

**Figure 3:**
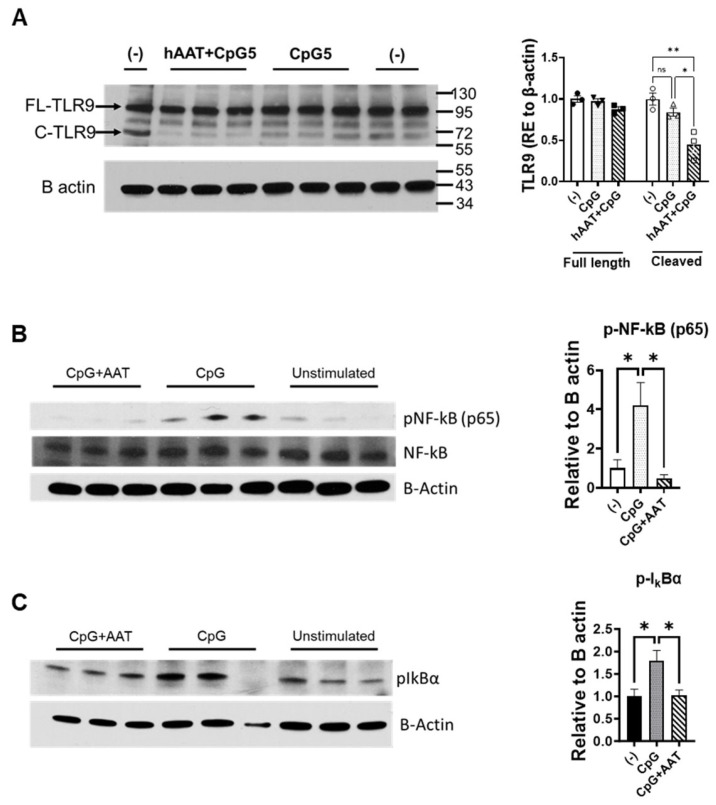
**The effect of hAAT on TLR9, p-NF-ĸB, and p-IĸBα.** DCs were treated with hAAT (1 mg/mL) and stimulated with or without 5 µg/mL CpG for 15 min. The cells were lysed and subjected to Western blot analyses using antibodies recognizing TLR9 (**A**), p-NF-kB (**B**), or p-IkBα (**C**). B-actin levels were used as loading controls; (-) and Unstimulated indicate untreated cells (with neither AAT nor CpG treatment). FL-TLR9, full-length TLR9; C-TLR9, cleaved TLR9; RE, relative to. n = 3 (cells from 3 mice) for all groups. *, *p* < 0.05 and **, *p* < 0.01 by one-way ANOVA. Western blot original images can be found in Figure S2.

TLRs require proteolytic processing in the endolysosome to initiate signaling in response to the ligands. Targeting this proteolytic processing may represent a novel strategy for inhibiting TLR-mediated pathogenesis. While hAAT is a protease inhibitor, the effect of hAAT on TLRs is unknown. Using Western blot analysis, we showed that hAAT treatment reduced the active form (cleaved) of TLR9 in DCs, indicating a novel mechanism of hAAT function in the immune system (Figure 3A and Figure S2). These results indicate a new mechanism underlying the inhibitory effect of hAAT on autoimmunity and inflammation.

As NF-kB is one of the center links for TLR mediated downstream signaling pathways, we further investigated the effect of hAAT on NF-kB-related factors. We treated cDCs with hAAT (1 mg/mL) and stimulated the cDCs with or without 5 µg/mL CpG for 15 min. The cells were lysed and subjected to Western blot analyses. We showed that hAAT treatment significantly reduced pNF-kB (p65) and p-IkBα levels (Figure 3B,C and Figure S2). These results indicate that the inhibitory effect of hAAT on DC activation and function is through its inhibitory effect on the NF-kB signaling pathway.

### 3.4. The Effect of AAT on TLR7 and TLR8 Pathways

**Figure 4:**
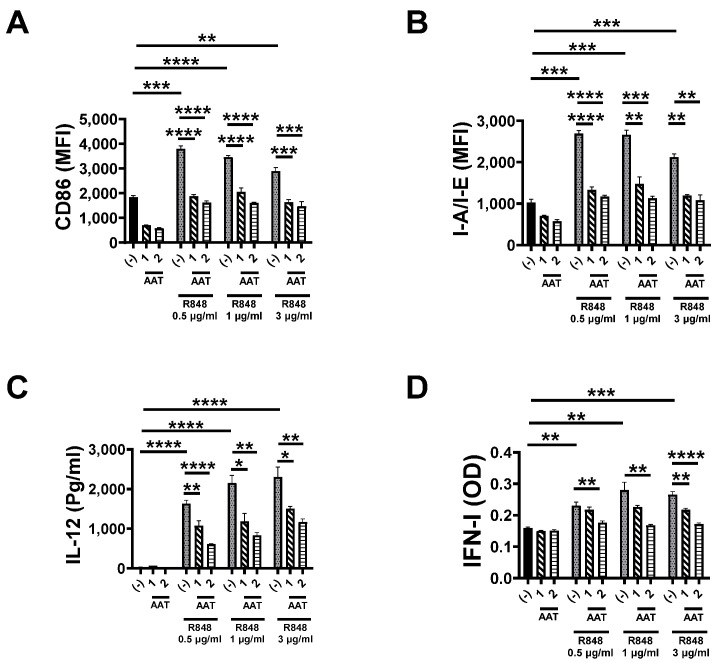
**The effect of hAAT on R848-induced DC activation and functions.** During the cDC induction, cells were treated with 0, 1, or 2 mg/mL hAAT. After the induction, cells were stimulated with R848 (0, 0.5, 1, or 3 µg/mL) for 6 h and subjected to flow cytometry analyses to detect DC activation using antibodies recognizing CD86 (**A**) and I-A/I-E (**B**). IL-12 (**C**) and INF-I (**D**) in the cell culture media were detected. n = 3 (cells from 3 mice) for all groups. *, *p* < 0.05; **, *p* < 0.01; ***, *p* < 0.001; and ****, *p* < 0.0001 by one-way ANOVA.

To investigate whether hAAT has any inhibitory effect on the TLR7 and TLR8 (TLR7/8) pathways, we used R848 (TLR7/8 agonist) to induce DC activation. As shown in Figure 4 and Figure S3, hAAT treatment significantly inhibited R848-induced DC activation (CD86 and I-A/I-E) and cytokine (IFN-I and IL-12) secretions. These results indicated that hAAT treatment also inhibits TLR7/8 pathways similarly to that on the TLR9 pathway.

### 3.5. The Effect of hAAT on Downstream Gene Expressions of TLR7/8

**Figure 5:**
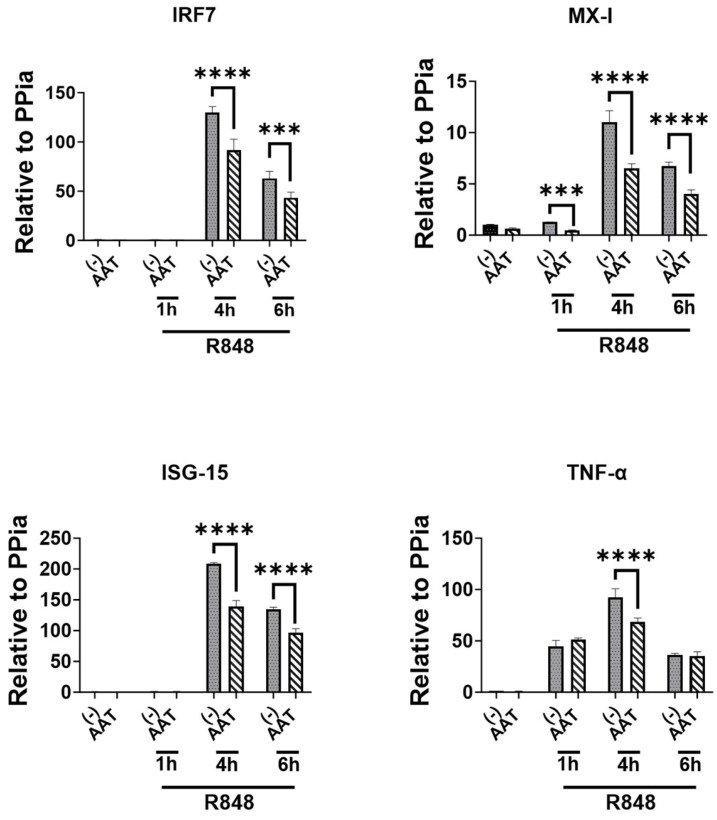
**The effect of hAAT on R848-induced gene expression.** Human-AAT-treated DCs were simulated with 0 or 1 μg/mL R848 for 1, 4, or 6 h. The cells were harvested and gene expressions were assessed by RT-qPCR using primers specific to genes of IRF-7, Mx-1, and ISG-15 or TNF-α. n = 3 to 8. ***, *p* < 0.001; and ****, *p* < 0.0001 by *t* test.

To extend our investigation, we treated DCs with or without 1 mg/mL hAAT and stimulated them with or without R848 (TLR7/8 agonist). We assessed the gene expression in these cells by RT-qPCR. As shown in Figure 5, hAAT significantly inhibited the expressions of IFN-I signature genes (IRF-7, Mx-1, and ISG-15) and TNF-α. These results show hAAT inhibitions on CpG-inducted DC activations and functions, demonstrating that hAAT has inhibitory effects on TLR7/8 and 9 pathways.

### 3.6. AAT-tg Mice Are Resistant to R848 Treatment

**Figure 6:**
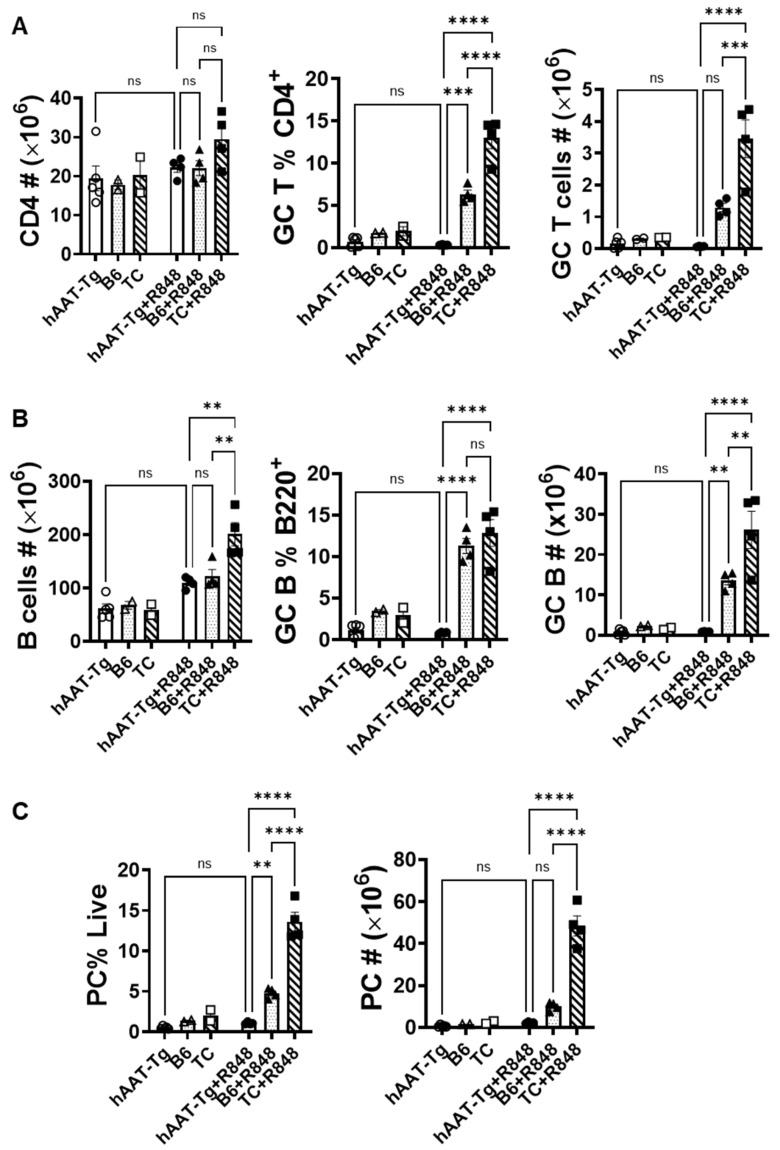
**Germinal center T cells (A), B cells (B), and plasma cells (C) in hAAT-tg mice are resistant to R848 treatment**. hAAT-tg, C57BL/6 (B6), and B6.TC (TC) mice were treated with 100 µL R848 in acetone 3 times (2-day interval). Control mice were treated with 100 µL acetone. n = 4 for all R848 treated groups. For untreated groups, n = 5 for hAAT-tg, n = 2 for B6, and n = 2 for TC. ns, *p* > 0.05; **, *p* < 0.01; ***, *p* < 0.001; and ****, *p* < 0.0001 by one-way ANOVA.

**Figure 7:**
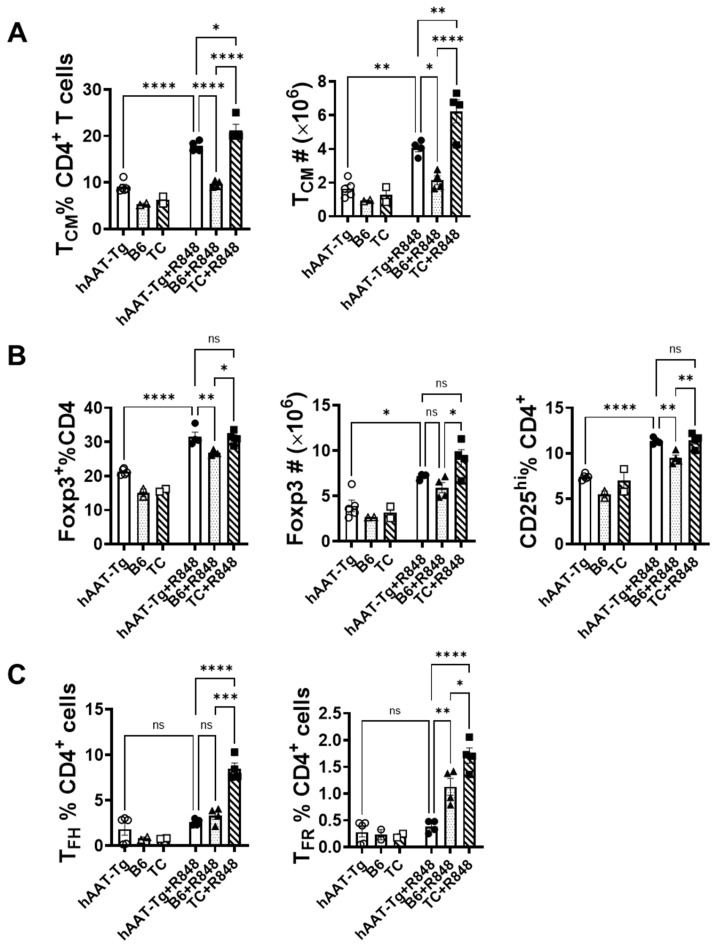
**The effect of hAAT on T cell populations.** (**A**). Central memory T cells (Tcm). (**B**). Treg related cells. (**C**). T follicular helper cells (T_FH_) and T follicular regulatory cells (T_FR_). n = 4 (cells from 4 mice) for all R848 treated groups. For untreated groups, n = 5 mice for hAAT-tg, n = 2 mice for B6, and n = 2 mice for TC. ns, *p* > 0.05; *, *p* < 0.05; **, *p* < 0.01; ***, *p* < 0.001; and ****, *p* < 0.0001 by one-way ANOVA.

In a previous study, we showed that splenocytes from hAAT-transgenic (hAAT-tg) mice are resistant to R848 induction in IL-6 and TNF-α productions [26]. To investigate this effect in vivo, we treated the hAAT-tg mice with or without 100 µL R848 for one week. In this experiment, we used age- and sex-matched C57BL/6 (B6) mice as a control and B6.Sle1.Sle2.Sle3 (B6.TC)-lupus-prone mice as a positive control. While the total numbers of B and T cells in hAAT-tg spleen were similar to that in B6 spleen, germinal center T, B, and plasma cells (PCs) in hAAT-tg mice were completely resistant to the R848 induction and was significantly lower than that in R484-treated B6 and B6.TC mice (Figure 6 and Figure S4). We dissected the effect of hAAT on T cell populations. As shown in Figure 7A,B, central memory T cells (Tcm) and Tregs in hAAT-tg mice were induced by R848 treatment, similarly to that in B6 and B6.TC mice. Importantly, the T follicular helper (TFH) and T follicular regulatory (TFR) cells in hAAT-tg mice were resistant to R848 induction, and this was significantly lower than that in B6 and B6.TC mice (Figure 7C). These results clearly demonstrate that hAAT blocks LR7/8 signaling in the germinal center and T follicular cells.

## 4. Discussion

Previous studies have shown that hAAT has therapeutic potential for autoimmune diseases including T1D, arthritis, and SLE [12,14,19,20,21,23,24,25,26]. However, the mechanisms underlying the protective effect of hAAT are not fully understood. Although it has been shown that hAAT inhibits CpG (TLR9 agonist)-induced DC activation and function [12,25], whether hAAT affects TLR7/8 signaling was not clear. In this study, we showed that hAAT inhibited TLR9 and 7/8 pathways similarly in a dose-dependent manner. These effects were clearly demonstrated by DC activation markers (CD80, CD86, I-A/IE), cytokine productions (IFN-I, IL-12), and the expressions of signature genes (TRF-7, MX-1, ISG-15) and the TNF-α gene. Intriguingly, these effects were observed shortly after the induction, indicating that hAAT affected the early steps of the signaling pathways. These findings provided critical evidence for further investigations of the mechanism of hAAT functions. In some conditions, we overserved that low-dose hAAT treatment potentiated cytokine secretion for yet unknown reasons. As hAAT has multiple functions (e.g., inhibiting proteinases and altering gene expressions) that may affect cytokine secretions differently in the low-dose condition, future study focusing on this condition may reveal new mechanisms.

The inhibitory effect of hAAT on NF-kB gene expression and function was previously overserved in islet cells, osteoclasts, senescent cells, and drosophila [15,34,35,36]. However, the direct effect of hAAT on TLR9-induced NF-kB activation remains unclear. Here, we showed that hAAT treatment significantly reduced the CpG-induced active form of NF-kB (phosphorylated, p-NF-kB). To further dissect the mechanism of the effect, we next focused on IK-Ba, which is a major inhibitor of NF-kB by binding to NF-kB. The phosphorylation of IK-Ba (p-IK-Ba) by IKK results in release of NF-kB for activation and the degradation of IK-Ba. We show that hAAT treatment significantly reduced CpG-induced p-IK-Ba levels. Consistent with the observation that hAAT treatment enhanced IK-Ba degradation [37], these results not only provided additional evidence for the inhibitory effects of hAAT, but also suggested that hAAT acted on further-upstream steps (IKK or TLR9) of TLR9 signaling.

As TLR7, 8, and 9 share a similar structure and require the proteolytic process for the efficient binding of their ligands (DNA or RNA) [6,9], we investigated the effect of hAAT on TLR9 activation. We showed that hAAT treatment significantly reduced the active (or cleaved) form of TLR9 in DCs, while the treatment had no effect on the levels of full-length TLR9. These results, for the first time, demonstrated that hAAT has a direct effect on the endosomal TLRs. It has been shown that the proteolytic processes of endosomal TLRs require multiple steps and enzymes including cathepsins, asparagine endopeptidase, and furin-like proprotein convertases [9,10,11]. While hAAT is a well-known serine proteinase inhibitor, it can inhibit other proteinases such as cathepsin K [15], caspase 3 [14], MMP-9 [38], and TNF-α converting enzyme (TACE or ADAM-17) [37]. It is possible that hAAT inhibited multiple enzymes in the proteolytic process of endosomal TLRs. Future studies evaluating the effects of hAAT on the enzymes activating LTR 7, 8, and 9 in different cell types will provide a better understanding of hAAT functions.

The germinal center (GC) is a special micro-structure in spleen and lymph nodes, and it plays important roles in the pathogenesis of autoimmune diseases, especially in B cell development [39]. After interacting with antigens presented by antigen presenting cells (APCs) and cognate CD4+ T cells, naïve B cells differentiate into memory B cells and GC B cells. In the GC, GC B cells receive help from cognate CD4+ T follicular helper cells (T_FH_) and undergo somatic hypermutation (SHM) and proliferation [40]. The T_FH_ in the GC can also promote the generation of long-lived plasma cells (PCs) through IL-21. GC B cells, in turn, present antigens to T_FH_ and stimulate IL-4 and IL-21 expression from T_FH_. In this study, we showed that mice overexpressing hAAT (hAAT-gt) are completely resistant to R848-induced GC B cells, GC T cells, T_FH_, T_FR_, and PCs, while control B6 and TC mice were very sensitive to the induction. These results clearly demonstrated that hAAT efficiently blocked TLR7/8-induced autoimmunity [41,42]. As intracellular TLRs are involved in the activation of APCs, T cells, and B cells [43,44], it is possible that hAAT inhibits all the processes involved these LTRs [45].

## 5. Conclusions

We demonstrated that hAAT inhibited TLR7/8- and 9-induced DC activation and function. Mechanistically, hAAT inhibits TLR proteolytic activation (or maturation). In the R848-induced mouse model, the transgenic expression of hAAT completely blocks the activation of B cells and T cell activation. These results provide a novel mechanism for the protective effects of hAAT in the control of autoimmunity.

## Figures and Tables

**Table 1:** **The primers used**.

	Forward	Reverse
Ppia	CACAGCCAAGGGTCGATTCC	CCCAGGTATCGTGCTTTGTCT
IL-12p40	AGCAGTAGCAGTTCCCCTGA	AGTCCCTTTGGTCCAGTGTG
IRF-7	CAGCGAGTGCTGTTTGGAGAC	AAGTTCGTACACCTTATGCGG
MX-I	GATCCGACTTCACTTCCAGATGG	CATCTCAGTGGTAGTCAACCC
ISG-15	GAGCTAGAGCCTGCAGCAAT	TAAGACCGTCCTGGAGCACT
TNF-a	CCACCACGCTCTTCTGTCTAC	AGGGTCTGGGCCATAGAACT

## Data Availability

The original contributions presented in the study are included in the article/supplementary material, further inquiries can be directed to the corresponding author.

## Supplementary Materials

The following supporting information can be downloaded at: https://www.mdpi.com/article/10.3390/biom15010043/s1, Figure S1: Late effect of hAAT on CpG-induced DC activation and functions. cDCs were induced from BM cells from B6 mice. During the induction, cells were treated with or without 0.1, 0.5, 1, and 2 mg/mL hAAT. After the induction, cells were stimulated with CpG (0, 1, 5, and 10 µg/mL) for 12 or 24 h and subjected to flow cytometry analyses to detect DC activation using antibodies recognizing CD80, CD86, and I-A/I-E (A–C). Cytokines (IFN-I, IL-12, and chemokine CXCL10) in culture media were detected (D–F). n = 3 (cell from 3 mice) for all groups. *, *p* < 0.05; **, *p* < 0.01; and ***, *p* < 0.001 by one-way ANOVA. Figure S2: Original Western blot images for Figure 3. Figure S3: Late effect of hAAT on R848-induced DC functions. During the cDC induction, cells were treated with 0, 0.1, 0.5, 1, or 2 mg/mL hAAT. After the induction, cells were stimulated with R848 (0, 0.5, 1, or 3 µg/mL) for 12 or 24 h. IL-12 in the cell culture media were detected. n = 3 mice for all groups. *, *p* < 0.05; **, *p* < 0.01; and ***, *p* < 0.001 by one-way ANOVA. Figure S4: Gating strategy for detecting cell populations in Figure 6 and Figure 7. Figure S5: Transgenic expression of hAAT on spleen weight and total numbers of splenocytes, B cells, and T cells. n = 4 mice for all R848 treated groups. For untreated groups, n = 5 mice for hAAT-tg, n = 2 mice for B6, and n = 2 mice for TC. *, *p* < 0.05; **, *p* < 0.01; and ***, *p* < 0.001. NS, *p* > 0.05 by one-way ANOVA.
