# Supplementary material for: The Inhibitory Effects of Alpha 1 Antitrypsin on Endosomal TLR Signaling Pathways
Source: Biomolecules. 2025 Jan 1;15(1):43. doi: 10.3390/biom15010043 (PMC11763108; doi:10.3390/biom15010043)
Supplement: Supplementary file 1 [file biomolecules-15-00043-s001.zip › biomolecules-3339838-supplementary.pdf]

Fig S1.

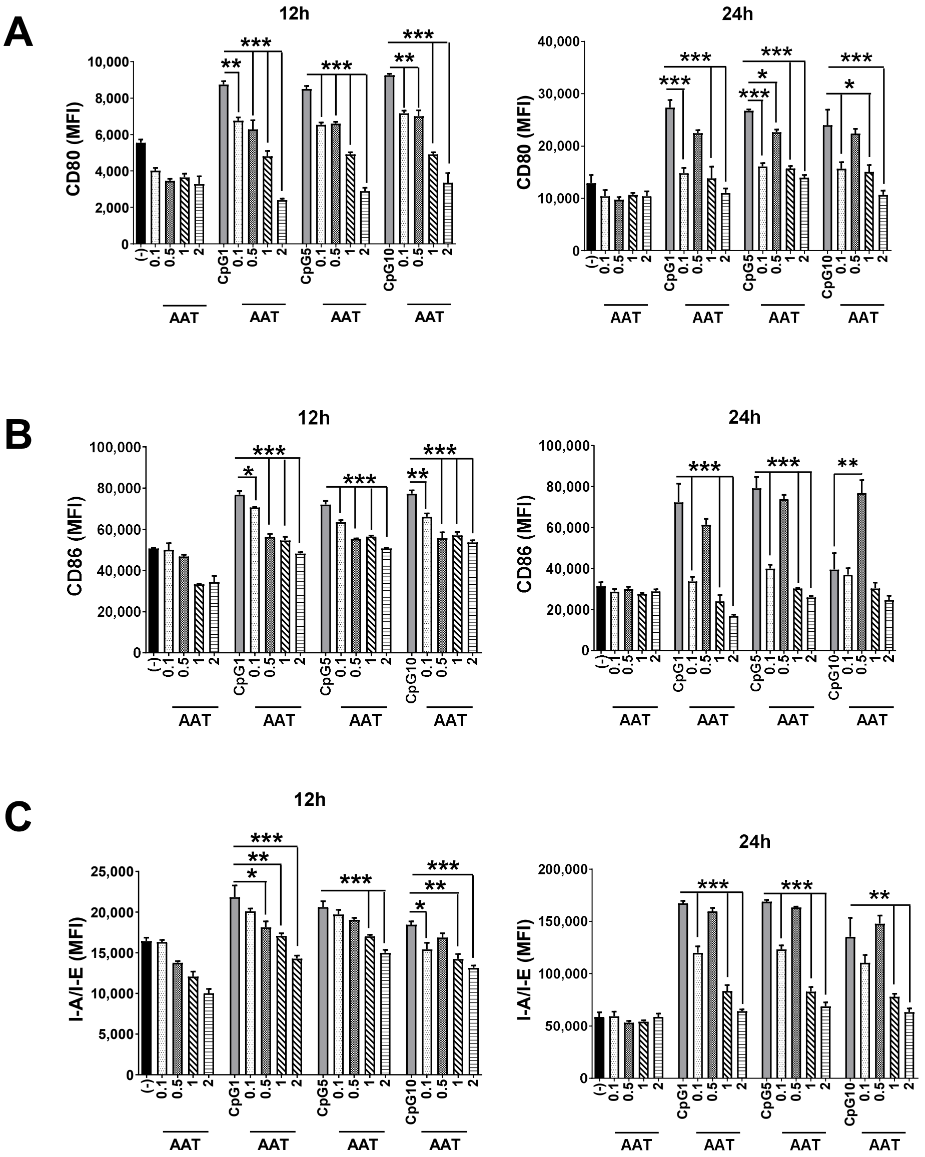
A
B
C
12h
24h
CD80 (MFI)
CD86 (MFI)
I-A/I-E (MFI)
(-)
0.1
0.5
1
2
CpG1
CpG5
CpG10
AAT
*
**
***
0
2,000
4,000
6,000
8,000
10,000
20,000
30,000
40,000
60,000
80,000
100,000
5,000
15,000
25,000
50,000
150,000
200,000

Fig S1.

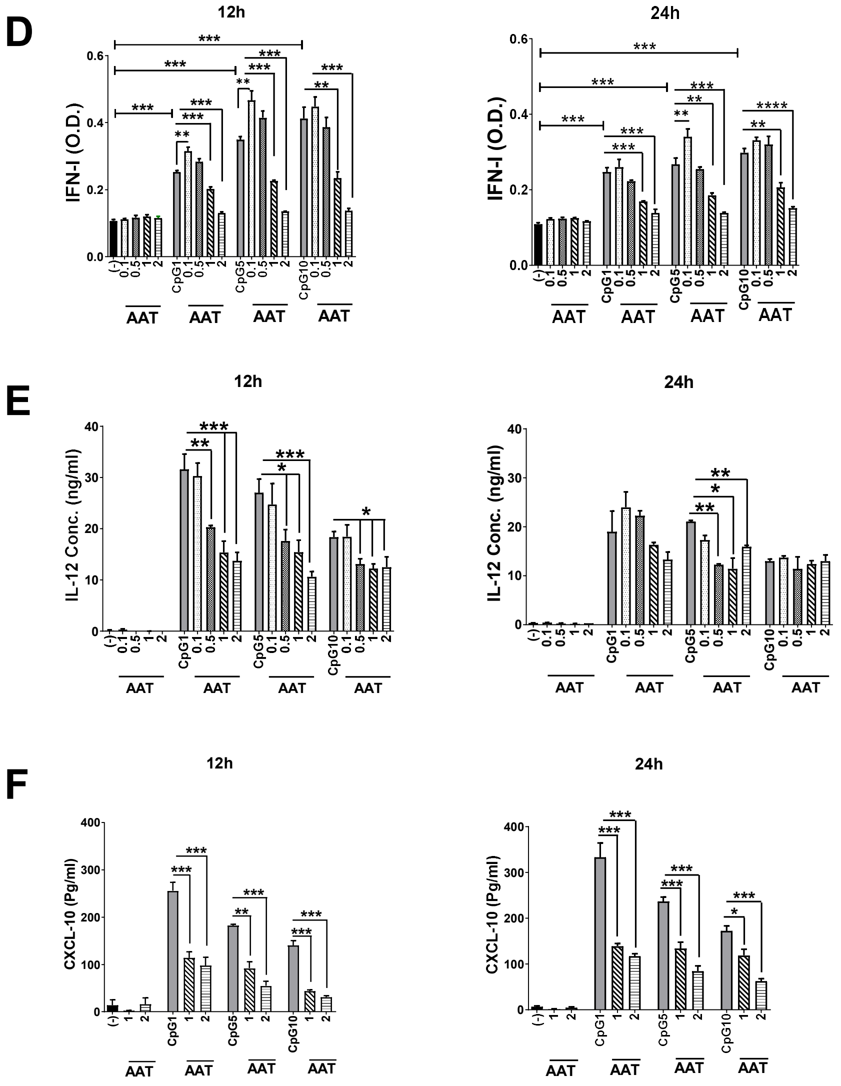
D
12h
24h
IFN-I (O.D.)
(-)
0.1
0.5
1
2
CpG1
CpG5
CpG10
AAT
E
12h
24h
IL-12 Conc. (ng/ml)
F
12h
24h
CXCL-10 (Pg/ml)

Fig S2.

A. TLR9

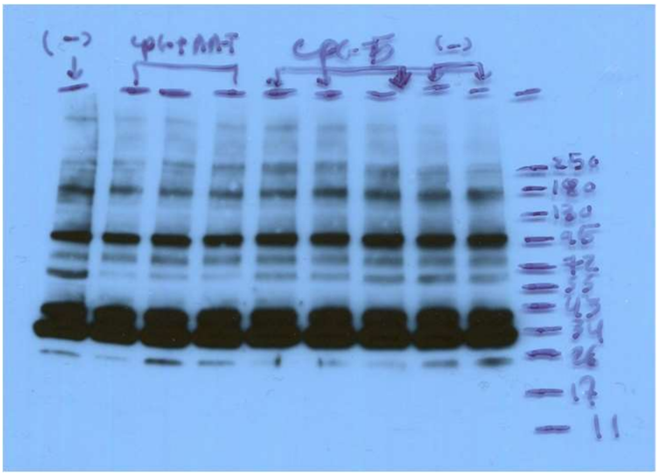

B. B-actin

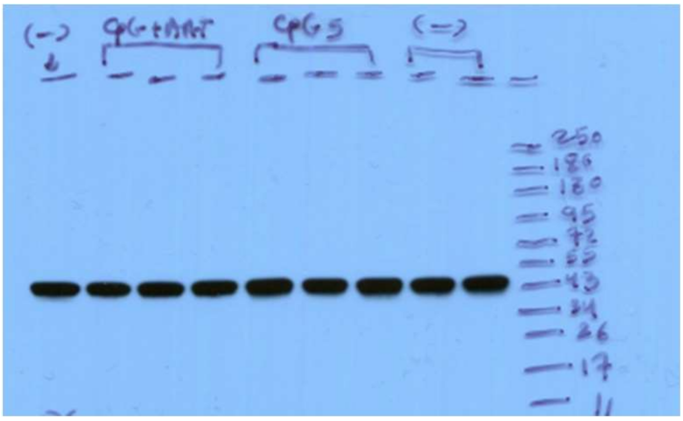

C. pNF-kB

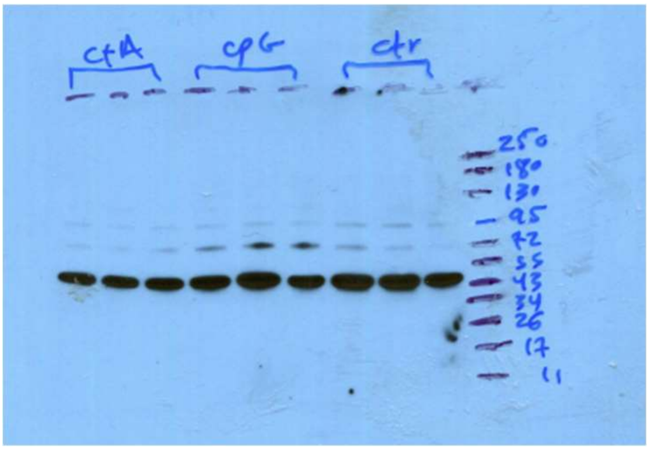

D. NF-kB

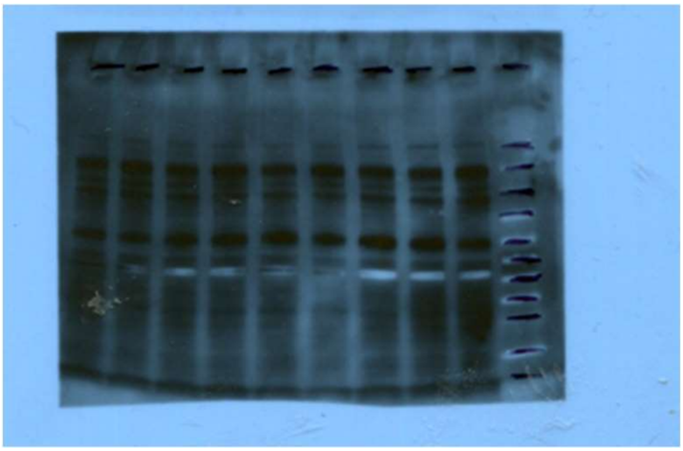

E. B-actin

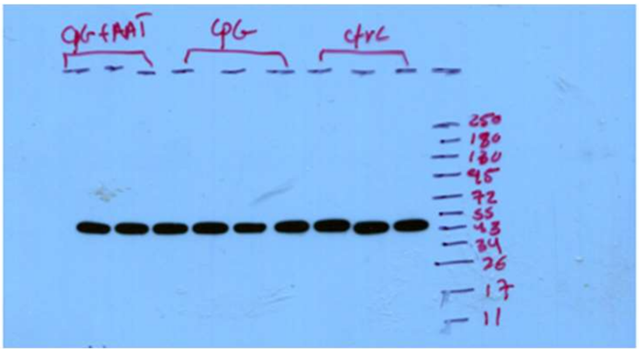

Fig S2.

F

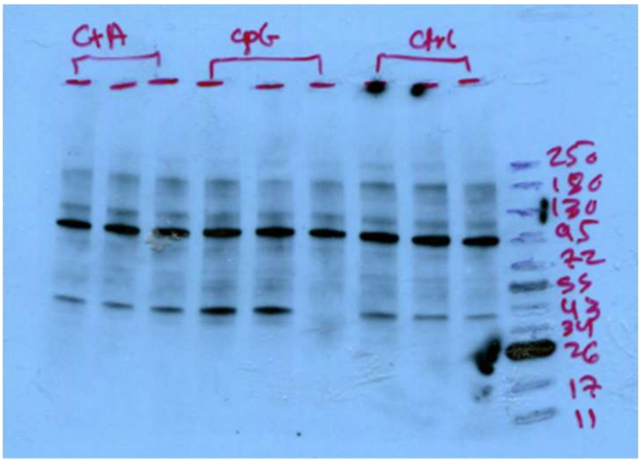

CtA
cpG
Ctrl
250
180
130
95
72
55
43
34
26
17
11

G

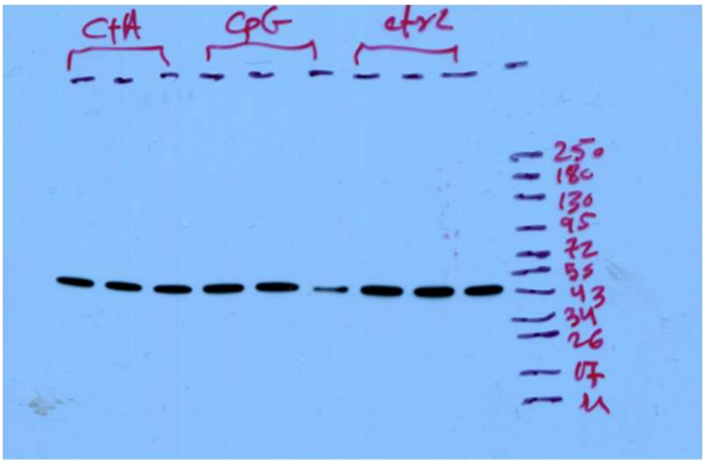

CtA
CpG
ctrl
250
130
95
72
55
43
34
26

Fig S3.

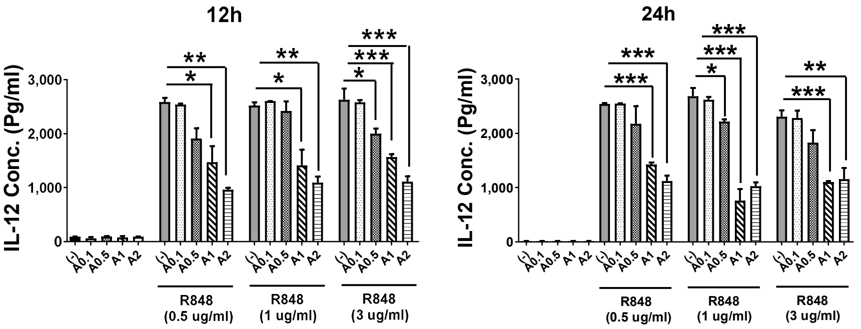
12h
24h
IL-12 Conc. (Pg/ml)
3,000
2,000
1,000
0
(-)
A0.1
A0.5
A1
A2
R848
(0.5 ug/ml)
R848
(1 ug/ml)
R848
(3 ug/ml)
*
**
***

Fig S4.

I. Gating strategy for T cells

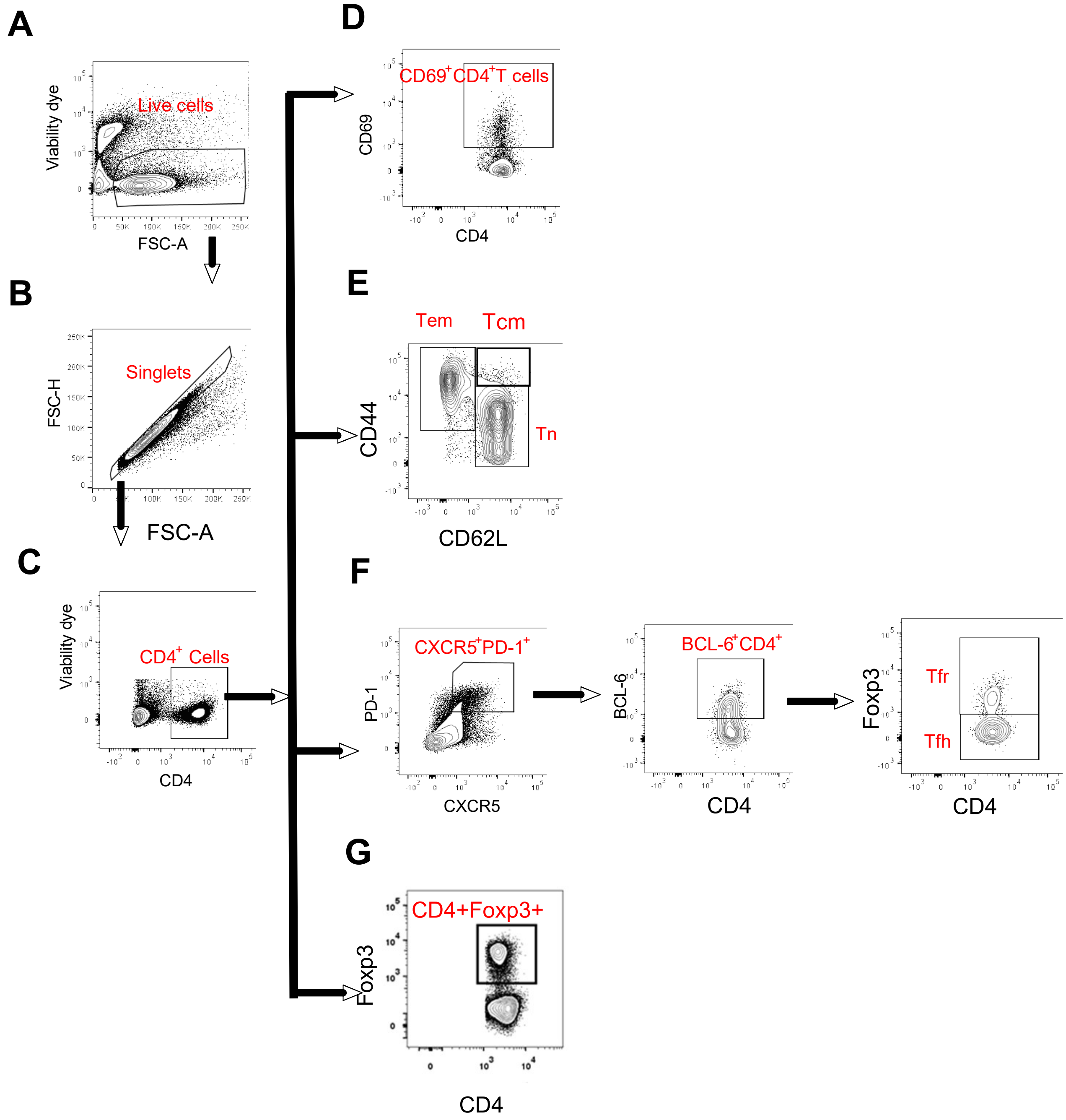

Fig S4.

II. Gating strategy for PC, GC B and GC T cells

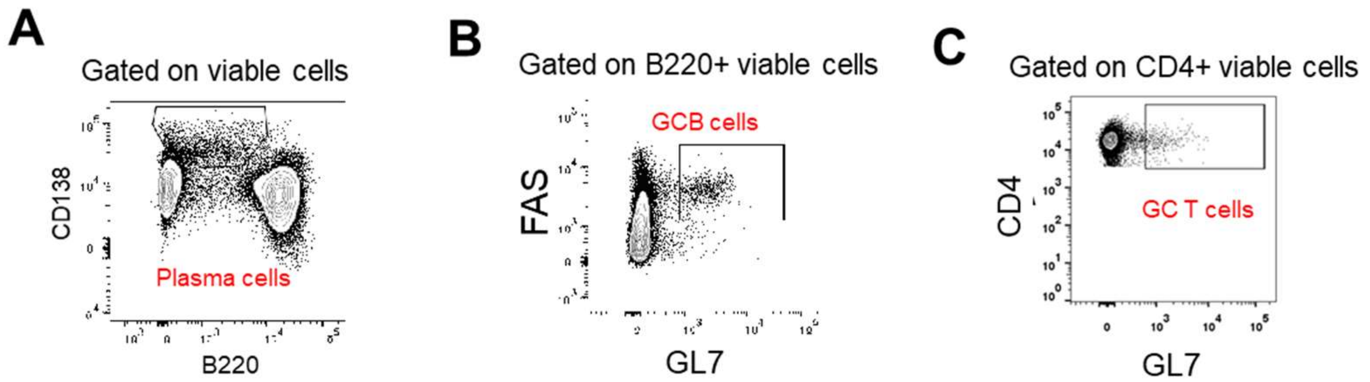

Fig S5.

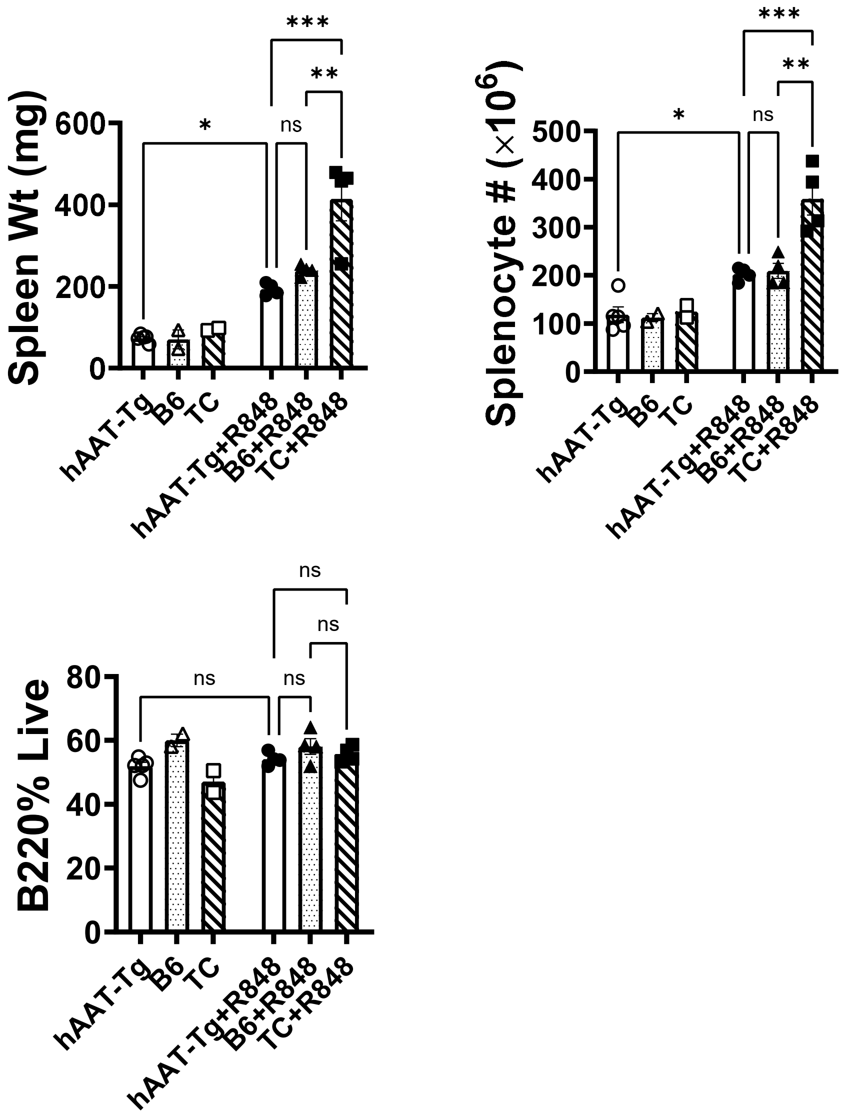
Spleen Wt (mg)
0
200
400
600
hAAT-Tg
B6
TC
hAAT-Tg+R848
B6+R848
TC+R848
*
ns
***
**
Splenocyte # (×10^6)
0
100
200
300
400
500
hAAT-Tg
B6
TC
hAAT-Tg+R848
B6+R848
TC+R848
*
ns
***
**
B220% Live
0
20
40
60
80
hAAT-Tg
B6
TC
hAAT-Tg+R848
B6+R848
TC+R848
ns
ns
ns
ns
